# Dysfunction of Mitochondrial Dynamics Induces Endocytosis Defect and Cell Damage in *Drosophila* Nephrocytes

**DOI:** 10.3390/cells13151253

**Published:** 2024-07-25

**Authors:** Jun-yi Zhu, Jianli Duan, Joyce van de Leemput, Zhe Han

**Affiliations:** 1 Center for Precision Disease Modeling, Department of Medicine, University of Maryland School of Medicine, Baltimore, MD 21201, USA; 2 Division of Endocrinology, Diabetes, and Nutrition, Department of Medicine, University of Maryland School of Medicine, Baltimore, MD 21201, USA

**Keywords:** mitochondria, Pink1, Parkin, Marf (MFN), endocytosis, reactive oxygen species (ROS), nephrocytes, *Drosophila*

## Abstract

Mitochondria are crucial for cellular ATP production. They are highly dynamic organelles, whose morphology and function are controlled through mitochondrial fusion and fission. The specific roles of mitochondria in podocytes, the highly specialized cells of the kidney glomerulus, remain less understood. Given the significant structural, functional, and molecular similarities between mammalian podocytes and *Drosophila* nephrocytes, we employed fly nephrocytes to explore the roles of mitochondria in cellular function. Our study revealed that alterations in the Pink1–Park (mammalian PINK1–PRKN) pathway can disrupt mitochondrial dynamics in *Drosophila* nephrocytes. This disruption led to either fragmented or enlarged mitochondria, both of which impaired mitochondrial function. The mitochondrial dysfunction subsequently triggered defective intracellular endocytosis, protein aggregation, and cellular damage. These findings underscore the critical roles of mitochondria in nephrocyte functionality.

## 1. Introduction

Mitochondria are the primary source of cellular ATP production [1], whose morphology and function are controlled by a balance between two opposing actions mitochondrial fusion and fission [2]. Mitochondrial fusion is controlled by Mitofusin 1 (MFN1) and Mitofusin 2 (MFN2) through the PTEN-induced kinase 1 (PINK1)–Parkin RBR E3 ubiquitin-protein ligase (PRKN) pathway; while mitochondrial fission is driven by Dynamin-related protein 1 (DRP1) [3,4,5]. Maintaining mitochondrial health is crucial, and the quality control and selective removal of damaged mitochondria play a tremendously important role in this process. Mitochondrial fission facilitates the isolation and removal of damaged mitochondria through mitophagy. Alongside regulating mitochondrial dynamics, the PINK1-PRKN pathway plays a crucial role in facilitating mitophagy to ensure the removal of damaged mitochondria, thus maintaining cellular health [6]. The dynamic regulation of mitochondrial fusion and fission plays a crucial role in maintaining cellular energy balance and ensuring mitochondrial health and functionality. Imbalances of mitochondrial dynamics have been associated with a range of diseases, including neurodegenerative disorders and metabolic diseases [7,8,9,10,11].

Kidneys are energy-demanding organs due to their constant filtering and reabsorption processes, and therefore highly dependent on mitochondria to produce ATP through cellular respiration, providing the necessary energy to support kidney functions [12,13,14]. Mitochondria are also involved in regulating pH levels within kidney cells and maintaining the balance of sodium, potassium, calcium, and other ions to ensure proper kidney function and fluid balance [15,16]. Furthermore, mitochondria facilitate the stability and dynamics of the podocyte cytoskeleton [17]. Podocytes are specialized cells in the glomeruli of the kidney, that are key to the filtration of blood to form urine and remove waste. Structural integrity provided by the cytoskeleton is crucial to the podocyte filtration function, which is dependent on elaborate foot processes that form interdigitating extensions with neighboring podocytes [18].

*Drosophila* pericardial nephrocytes, referred to here as nephrocytes, have striking structural and functional similarities to mammalian podocytes [19,20,21,22]. Both cells form highly specialized structures called slit diaphragms, which along with the basement membrane act as size- and charge-dependent filtration barriers [19]. The mitochondrial Coenzyme Q (CoQ) pathway plays a key role in the electron transport chain to efficiently produce ATP [23]. Deficiency for CoQ2, a component of the CoQ pathway, led to a decline in nephrocyte function, mitochondrial dysfunction, and abnormal localization of the slit diaphragm in *Drosophila* nephrocytes. Either expressing the human COQ2 gene or dietary supplementation with coenzyme Q10, the product of the CoQ pathway, restored these nephrocyte defects [23]. This and other studies [24,25] underscored the value of the *Drosophila* nephrocyte as a model system for studying mitochondrial functions.

Here, we used *Drosophila* to explore the roles of mitochondria in nephrocytes in vivo. Genetic modification of mitochondrial dynamic control induced fragmented or enlarged mitochondria, either of which resulted in reduced mitochondrial function. The dysfunctional mitochondria caused defective intracellular endocytosis, and protein aggregation, and culminated in cellular damage and filtration dysfunction. 

## 2. Materials and Methods

### 2.1. Drosophila Lines

All fly stocks were maintained at 25 °C and fed a standard diet (Meidi Laboratories, Baltimore, MD, USA). *Drosophila* stocks were sourced from the Bloomington Drosophila Stock Center (Indiana University Bloomington, Bloomington, IN, USA). The following lines were utilized in the experiments: *Dot*-Gal4 (ID 6903), UAS-mito-GFP (ID 8442), UAS-*Pink1*-RNAi (ID 31170 and 41671), UAS-*park*-RNAi (ID 31259 and 37509), UAS-*Marf*-RNAi (ID 31157 and 55189), UAS-*Pink1*-OE (ID 51648), UAS-*park*-OE (ID 51651), UAS-*Marf*-OE (ID 67157), UAS-*Rab5*-RNAi (ID 30518 and 34832), UAS-*Rab5*-YFP (ID 24616), UAS- *Rab7*-YFP (ID 23270), UAS-*Rab11*-YFP (ID 9790). *w*^1118^ (ID 3605) flies served as control. 

### 2.2. Mitochondrial Membrane Potential Measurement in Drosophila Nephrocytes

The mitochondrial membrane potential was measured using a tetramethylrhodamine, methyl ester (TMRM) assay kit (Abcam, Cambridge, UK; ab228569). Nephrocytes were dissected from 4-day-old adult female flies and maintained at room temperature in artificial hemolymph [24]. Cells were incubated with TMRM (1 µg/mL; Invitrogen, Waltham, MA, USA) for 1 h and subsequently washed three times with phosphate-buffered saline (1XPBS) containing 0.1% Triton X-100 (Sigma-Aldrich, St. Louis, MO, USA) (PBST). Mitochondrial membrane potential was determined based on TMRM fluorescence levels in nephrocytes, measured using fluorescence confocal microscopy in live cells (ZEISS LSM 900; see details below). 

### 2.3. Transmission Electron Microscopy (TEM) for Drosophila Nephrocytes

Nephrocytes were dissected from 4-day-old adult female flies and maintained in artificial hemolymph. The cells were initially fixed with 8% paraformaldehyde in 1XPBS for 10 min, then transferred to a fixation buffer containing 4% paraformaldehyde and 2.5% glutaraldehyde. Subsequent processing and analysis of the cells were conducted [26] using an FEI Tecnai T12 TEM at the Electron Microscopy Core Imaging Facility at the Center for Innovative Biomedical Resources (CIBR) (University of Maryland School of Medicine, Baltimore, MD, USA).

### 2.4. Measurement of Mitochondrial Dimensions 

To measure the mitochondrial size in nephrocytes, we utilized *Dot*-Gal4, UAS-mito-GFP fly lines crossed with the overexpression (OE), or RNAi (IR) lines targeting Pink1, park, or Marf. Images were conducted using ZEISS LSM900 and analyzed with ImageJ software [27] (National Institutes of Health, Bethesda, MD, USA; version 1.52a). Each mitochondrion was manually outlined using the Freehand selection tool and measured. The average size of mitochondria was calculated based on 30 mitochondria from a single nephrocyte. For quantitation, a total of 30 nephrocytes per genotype were analyzed, derived from six 4-day-old adult female flies. 

### 2.5. Assessment of Dextran and Albumin Uptake in Drosophila Nephrocytes

Ex vivo assays measuring dextran and albumin uptake were conducted to evaluate the filtration function of nephrocytes. Nephrocytes were dissected from 4-day-old adult female flies and maintained in artificial hemolymph. Cells were incubated with dextran, Texas Red, 10,000 MW (0.05 mg/mL; Invitrogen, D1828) or albumin, Alexa Fluor 488 (0.05 mg/mL; Invitrogen, A13100) for 20 min, followed by fixation with 4% paraformaldehyde in 1XPBS (Thermo Fisher Scientific, Waltham, MA, USA) for 10 min. The uptake capacity of dextran and albumin was determined by the fluorescence levels in the nephrocytes, which were assessed using ZEISS. 

### 2.6. Fluorescent Immunochemistry

Four-day-old adult female flies were dissected and heat-fixed for 20 s in 100 °C artificial hemolymph to prepare them for immunohistochemistry. Primary antibodies used included a mouse monoclonal anti-Pyd antibody (PYD2) sourced from Developmental Studies Hybridoma Bank (DSHB; University of Iowa, Iowa City, IA, USA) at 1:100 dilution in PBST; and, a mouse monoclonal anti-ubiquitinylated proteins antibody, clone FK2 (Enzo Life Sciences, Farmingdale, NY, USA) at 1:500 dilution in PBST. Secondary antibodies, Alexa Fluor 555 (Thermo Fisher Scientific) and Alexa Fluor 488 (Thermo Fisher Scientific), were each used at 1:1000 dilution in PBST. DAPI (Thermo Fisher Scientific) was utilized to visualize the nuclei at a dilution of 1:1000 in PBST. The nephrocytes were washed three times with PBST, and blocked in PBST containing 2% bovine serum albumin (BSA; Sigma-Aldrich) for 30 min, followed by overnight incubation with primary antibodies at 4 °C. The following day, cells were washed three times with 1XPBST, incubated with secondary antibodies and DAPI at room temperature for 2 h, then washed three times with 1XPBST, and mounted with Vectashield mounting medium (Vector Laboratories, Malvern, PA, USA). Images were captured using ZEISS LSM 900.

### 2.7. Reactive Oxygen Species Assay in Drosophila Nephrocytes

Levels of reactive oxygen species (ROS) were measured with a dihydroethidium (DHE) assay kit (Invitrogen, D11347). Nephrocytes were dissected from 4-day-old adult female flies and maintained in artificial hemolymph. The cells were incubated with DHE (1 µg/mL; Invitrogen) for 30 min and subsequently washed three times with PBST. The ROS levels were determined based on the DHE fluorescence levels in the nuclei of the nephrocytes, using ZEISS LSM 900. 

### 2.8. Fluorescence Confocal Microscopy of Drosophila Nephrocytes

Imaging for dextran uptake, albumin uptake, UAS-mito-GFP, UAS-*Rab5*-YFP, UAS-*Rab7*-YFP, UAS-*Rab11*-YFP fly strains, fluorescent immunocytochemistry for anti-FK2, DHE, and DAPI was carried out confocal technology; a ZEISS LSM900 microscope with a 20× Plan-Apochromat 0.8 N.A. air objective (ZEN Blue, edition 3.0, acquisition software). Fluorescent immunocytochemistry for anti-Pyd and TMRM and fluorescent imaging for the UAS-mito-GFP fly strains were carried out using a ZEISS LSM900 microscope with a 63× Plan-Apochromat 1.4 N.A. oil objective under Airyscan SR mode (ZEN Blue, Zeiss, Jena, Germany; edition 3.0, acquisition software). For quantitative comparison of intensities, uniform settings were selected to prevent oversaturation and were consistently applied across all images captured for that assay. ImageJ Software [27] (version 1.52a) was used for image processing.

### 2.9. Statistical Analysis of Drosophila Assays

Statistical analyses were conducted using PAST.exe software (Natural History Museum, Norway; version 2.17C). Initially, data were assessed for normality using the Shapiro–Wilk test (α = 0.05). Data with normal distribution were then analyzed by the Kruskal–Wallis H-test followed by a Dunn’s test for comparisons between multiple groups. For quantitation, 30 nephrocytes per genotype were examined from 6 adult female flies. The results are presented as mean ± SD. Statistical significance (*) was defined as *p* < 0.05.

## 3. Results

### 3.1. Mitochondria Highly Existed in Drosophila Nephrocytes

**Figure 1:**
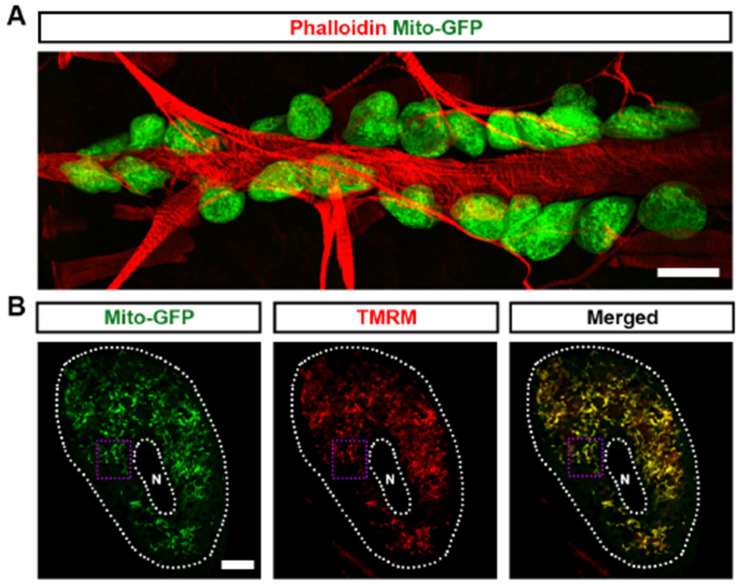

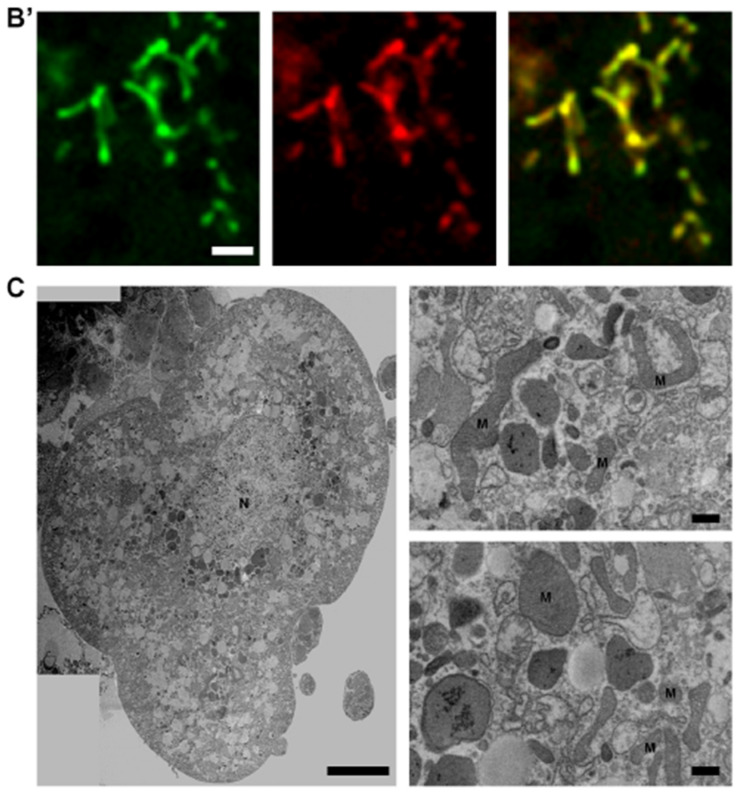
Mitochondria in *Drosophila* nephrocytes: (**A**) UAS-mito-GFP (green) induced by nephrocyte-specific driver *Dot*-Gal4 to label the mitochondria in *Drosophila* nephrocytes (*Dot* > mito-GFP, female, 4-day-old). Phalloidin (red) labels the actin filaments and was used to visualize the fly heart. Scale bar = 20 µm. (**B**,**B’**) *Dot* > mito-GFP (green) was to label the mitochondria in *Drosophila* nephrocytes (female, 4-day-old). Mitochondrial membrane potential was indicated by tetramethylrhodamine, methyl ester (TMRM; red). White dotted lines, outline the nephrocyte and the nucleus (N); purple dotted box, outlines the area magnified in (**B’**). Scale bar: (**B**) = 5 µm; (**B’**) = 1 µm. (**C**) Mosaic of transmission electron microscopy (TEM) images showing nephrocyte ultrastructure with nucleus (N) and mitochondria (M). Scale bars: (left) = 5 µm; (right, both) = 2 µm.

Mitochondria are essential organelles that are responsible for oxidative phosphorylation, the primary mechanism of cellular ATP production [28,29]. Mitochondrial function is closely tied to dynamic changes in size and shape, which are regulated by processes of fission and fusion [30]. To investigate the role of mitochondria in *Drosophila* nephrocyte function, we employed the UAS-GAL4 system combined with UAS-mito-GFP, which specifically labels the mitochondria to visualize their morphology. We found that mitochondria are highly abundant in *Drosophila* nephrocytes (Figure 1A). The mitochondria showed both round and elongated shapes, indicative of mitochondrial dynamics (Figure 1B,B’). Mitochondrial membrane potential is a critical component for ATP production through oxidative phosphorylation [31]. We used tetramethylrhodamine methyl ester (TMRM) to detect mitochondrial membrane potential as a measure of mitochondrial function in the *Drosophila* nephrocytes. We observed that TMRM was highly expressed and co-located with mitochondria in the nephrocytes, indicative of highly active mitochondria (Figure 1B,B’). Imaging by transmission electron microscopy (TEM) supported the presence of both round and elongated mitochondria in the nephrocytes (Figure 1C). Overall, the data show that mitochondria are abundantly present in nephrocytes with signs of the expected dynamics and activity. 

Regulation of mitochondrial dynamics in Drosophila nephrocytes by the Pink1–Park pathway. Mitochondrial dynamics are partially regulated by the Pink1–Park pathway, *Drosophila* ortholog of the human PINK1–PRKN pathway, which also regulates mitophagy to eliminate damaged mitochondria [4]. Consequently, we proceeded to investigate whether the genetic modification of the Pink1–Park pathway in *Drosophila* nephrocytes influences mitochondrial morphology and function. To achieve this, we combined the nephrocyte-specific driver *Dot*-Gal4 with either RNAi knockdown (UAS-*Pink1*-IR, UAS-*park*-IR, or UAS-*Marf*-IR) or overexpression (UAS-*Pink1*-OE, UAS-*park*-OE, or UAS-*Marf*-OE) of *Pink1*, *park* or *Marf* in flies. Two independent RNAi lines were examined for each gene, yielding consistent results. Therefore, representative data from one line are displayed in the figures

**Figure 2:**
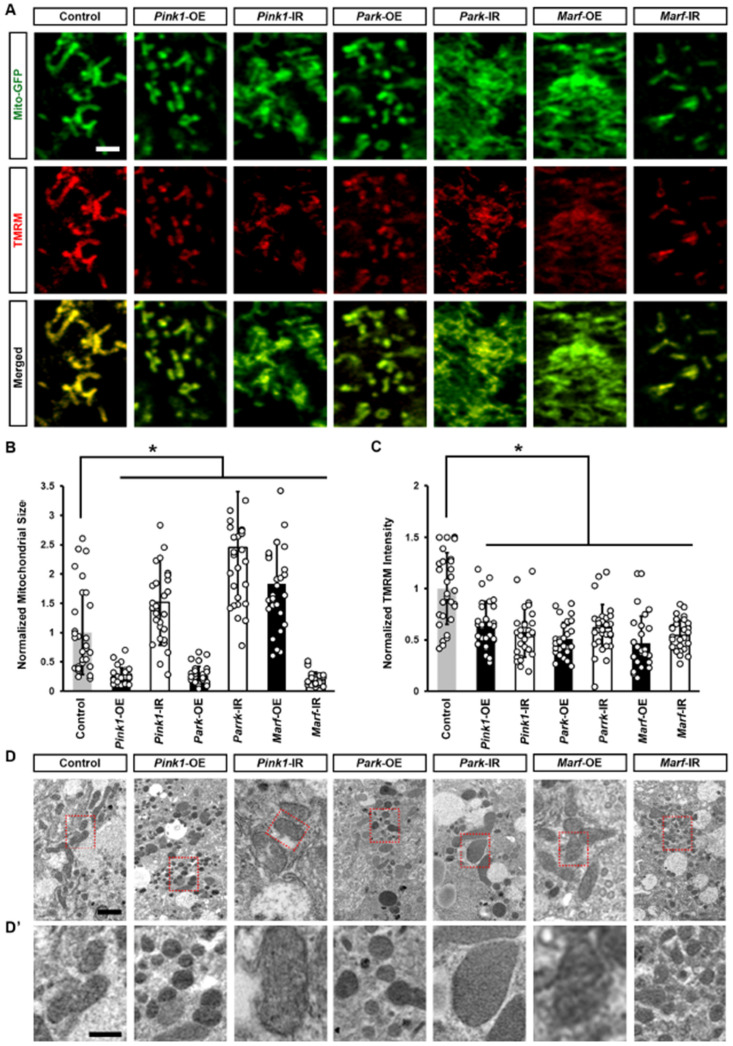
Defective mitochondrial dynamics resulted in altered mitochondrial morphology and reduced mitochondrial function: (**A**–**D’**) Mitochondrial morphology in *Drosophila* nephrocytes (female, 4-day-old) overexpressing (OE) or inhibiting (RNAi; IR) *Pink1*, *park*, or *Marf*. Control, *Dot* > mito-GFP. (**A**) *Dot* > mito-GFP (green) was to label the mitochondria. Mitochondrial membrane potential was indicated by TMRM (red). Scale bar = 1 µm. (**B**) Quantitation of mitochondrial size in *Pink1*/*park*/*Marf*-OE/IR nephrocytes. (**C**) Quantitation of TMRM fluorescence in *Pink1*/*park*/*Marf*-OE/IR nephrocytes. (**D**,**D’**) Transmission electron microscopy (TEM) of mitochondria in nephrocytes. Red dotted box outlines the area magnified in (**D’**). Scale bar: (**D**) = 5 µm; (**D’**) = 2 µm. N = 30 mitochondria from 6 flies, per group per assay. Results presented as mean ± SD; statistical significance: *, *p* < 0.05.

We first examined the mitochondrial morphology of nephrocytes from the progenies. *Pink1* or *park* overexpression, or *Marf* silencing changed mitochondrial morphology and significantly reduced mitochondria size; while the opposite was true too, *Pink1* or *park* silencing, or *Marf* overexpression changed mitochondrial morphology and significantly increased mitochondria size (Figure 2A,B,D,D’). These findings indicate altered mitochondrial fission–fusion dynamics, which were reflected in significantly reduced mitochondrial membrane potential (Figure 2A,C), indicating mitochondrial dysfunction. The data implicate defective mitochondrial dynamics and reduced mitochondrial function when the Pink1–Park pathway is dysregulated, supporting its importance in nephrocyte mitochondrial function. 

### 3.2. Pink1–Park-Mediated Defective Mitochondrial Dynamics Lead to Reduced Nephrocyte Function and a Disrupted Slit Diaphragm Filtration Structure

**Figure 3:**
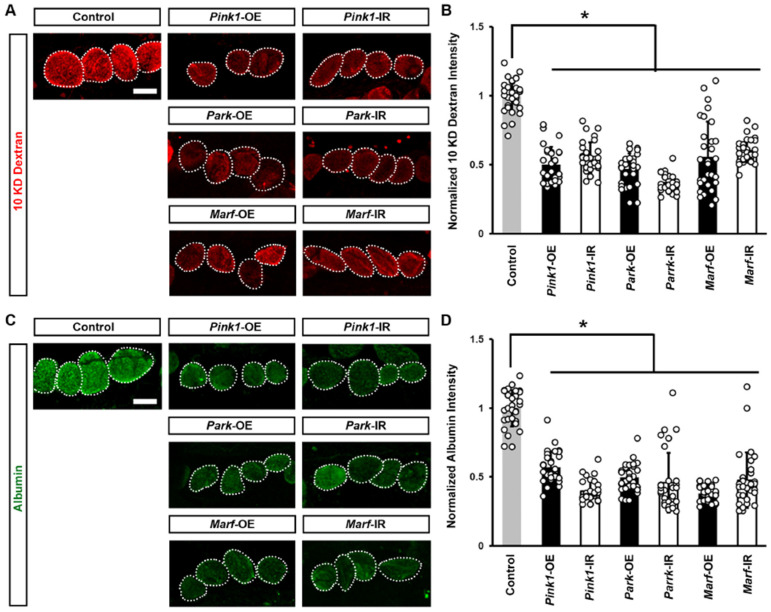
Defective mitochondrial dynamics reduced nephrocyte function: (**A**–**D**) Function in *Drosophila* nephrocytes (female, 4-day-old) overexpressing (OE) or inhibiting (RNAi; IR) *Pink1*, *park*, or *Marf*. Control, *Dot* > *w*^1118^. (**A**) 10 kDa dextran (red) uptake by nephrocytes. Scale bar = 25 µm. (**B**) Quantitation of 10 kDa dextran fluorescence in *Pink1*/*park*/*Marf*-OE/IR nephrocytes, relative to control. (**C**) Albumin (green) uptake by nephrocytes. Scale bar = 25 µm. (**D**) Quantitation of albumin fluorescence in *Pink1*/*park*/*Marf*-OE/IR nephrocytes, relative to control. N = 30 nephrocytes from 6 flies, per group per assay. Results presented as mean ± SD; statistical significance: *, *p* < 0.05.

Next, we examined changes in the function of *Drosophila* nephrocytes associated with the altered mitochondrial morphology. We measured nephrocyte filtration function with an ex vivo assay of cell ability to uptake the smaller 10 kDa dextran or the larger molecular weight albumin fluorescent particles. We noted a significant decrease in both 10 kDa dextran and albumin intensity when silencing or overexpressing the Pink1–Park pathway genes in the nephrocytes (Figure 3), indicating nephrocyte functional decline. 

**Figure 4:**
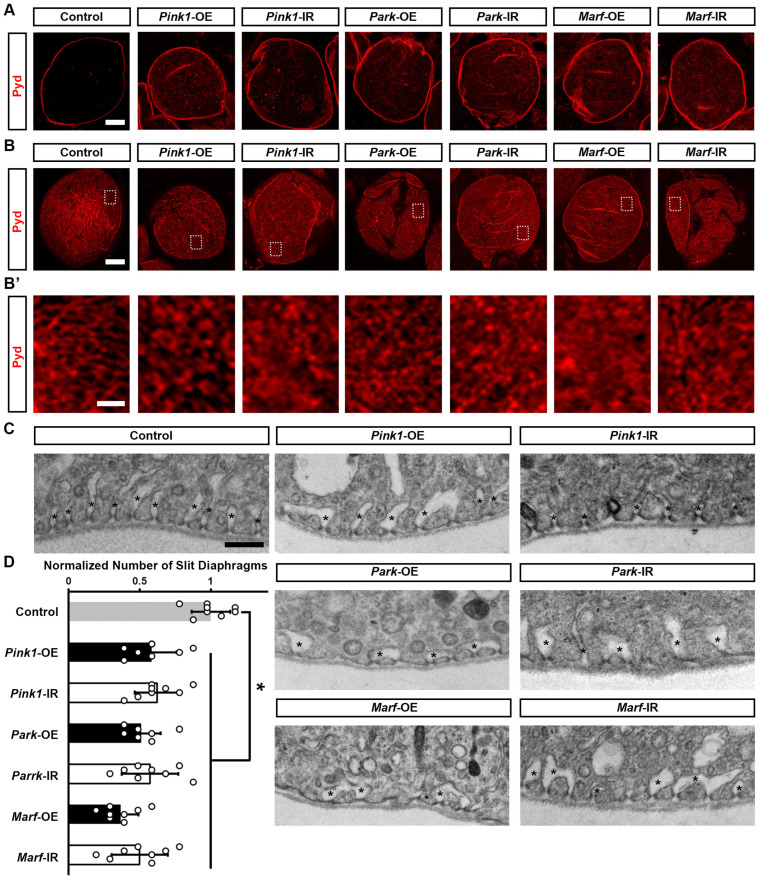
Defective mitochondrial dynamics in nephrocytes lead to a disrupted slit diaphragm structure: (**A**–**C**) Slit diaphragm in *Drosophila* nephrocytes (female, 4-day-old) overexpressing (OE) or inhibiting (RNAi; IR) *Pink1*, *park*, or *Marf*. Control, *Dot* > *w*^1118^. (**A**–**B’**) Distribution of slit diaphragm protein Polychaetoid (Pyd; red) by immunofluorescence in *Drosophila* nephrocytes. (**A**) Imaged at medial optical sections. Scale bar = 5 µm. (**B**,**B’**) Imaged at nephrocyte surface. While the dotted box outlines the area magnified in (**B’**). Scale bar: (**B**) = 5 µm; (**B’**) = 1 µm. (**C**) Transmission electron microscopy (TEM) displaying nephrocyte ultrastructure featuring slit diaphragms and lacunar channels around the circumference. Asterisk (*) indicates lacunar channel. Scale bar = 200 nm. (**D**) Quantitation of numbers of slit diaphragms in *Pink1*/*park*/*Marf*-OE/IR nephrocytes, relative to control. N = 8 flies, per group. Results presented as mean ± SD; statistical significance: *, *p* < 0.05.

To understand how altered mitochondrial dynamics led to defective nephrocyte uptake function, we examined the slit diaphragm and lacunar channel structures, which are crucial for the filtration function of nephrocytes [32,33]. Immunochemistry for the slit diaphragm protein Polychaetoid (Pyd) in control nephrocytes revealed its typical localization as a well-defined and continuous circumferential ring (Figure 4A, medial optical section) and a uniform and smoothly distributed fingerprint-like pattern on the surface (Figure 4B,B’). Genetically modified Pink1–Park pathway by either silencing or overexpressing *Pink1*, *park*, or *Marf* in the nephrocytes disrupted nephrocyte Pyd localization; such that a significant portion of the Pyd protein is internalized rather than remaining on the surface (Figure 4A). We also observed a disrupted slit diaphragm localization pattern at the nephrocyte surface, no longer resembling a clear fingerprint-like pattern (Figure 4B,B’). To visualize the lacunar channel ultrastructure in *Drosophila* nephrocytes, we used a transmission electron microscope (TEM). Control nephrocytes showed regularly spaced and shaped lacunar channels along the circumference of the cell (Figure 4C), whereas channel shapes were severely disrupted and the numbers of slit diaphragms were significantly declined in nephrocytes after *Pink1*, *park*, or *Marf* were silenced or overexpressed (Figure 4C,D). Together these data indicate that genetic modification of Pink1–Park-mediated defective mitochondrial dynamics leads to disrupted filtration structures and associated dysfunction in nephrocytes.

### 3.3. Pink1–Park-Mediated Defective Mitochondrial Dynamics Lead to Impaired Endocytic Membrane Trafficking 

**Figure 5:**
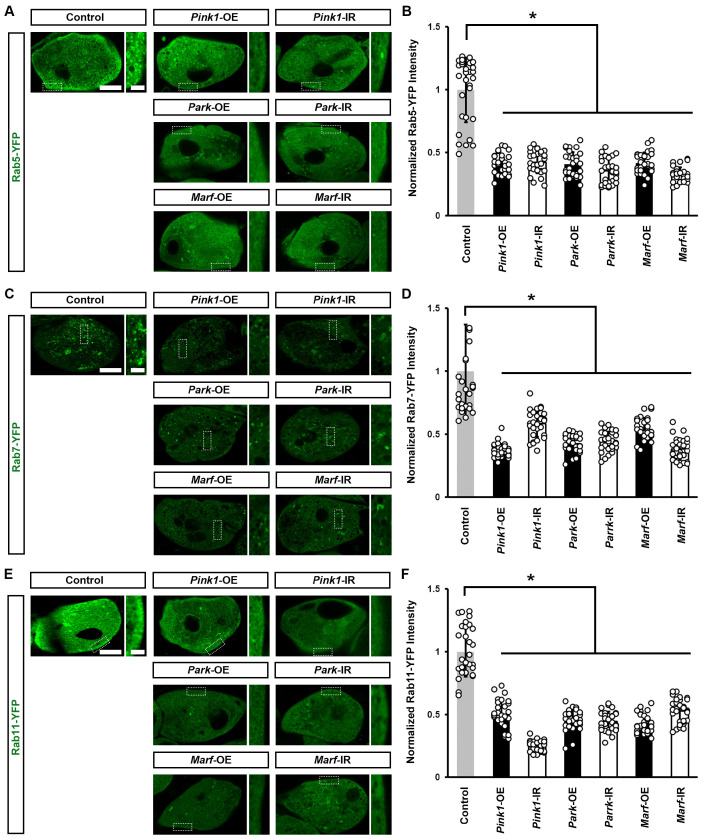
Defective mitochondrial dynamics in nephrocytes impaired endocytic membrane trafficking: (**A**–**F**) Endocytic membrane trafficking in *Drosophila* nephrocytes (female, 4-day-old) overexpressing (OE) or inhibiting (RNAi; IR) *Pink1*, *park* or *Marf*. Control, (**A**) *Dot* > Rab5-YFP, (**B**) *Dot* > Rab7-YFP (**C**) *Dot* > Rab11-YFP. (**A**) Early endosome visualized by Rab5-YFP (green) in nephrocytes. Scale bar = 5 µm. Boxed areas are shown magnified to the right (scale bar: 1 μm). (**B**) Quantitation of Rab5-YFP fluorescence in *Pink1*/*park*/*Marf*-OE/IR nephrocytes. (**C**) Late endosomes visualized by Rab7-YFP (green) in nephrocytes. Scale bar = 5 µm. Boxed areas are shown magnified to the right (scale bar: 1 μm). (**D**) Quantitation of Rab7-YFP fluorescence in *Pink1*/*park*/*Marf*-OE/IR nephrocytes. (**E**) Recycling endosomes visualized by Rab11-YFP (green) in nephrocytes. Scale bar = 5 µm. Boxed areas are shown magnified to the right (scale bar: 1 μm). (**F**) Quantitation of Rab11-YFP fluorescence in *Pink1*/*park*/*Marf*-OE/IR nephrocytes, relative to control. N = 30 nephrocytes from 6 flies, per group per assay. Results presented as mean ± SD; statistical significance: *, *p* < 0.05.

We previously found that endocytic membrane trafficking is essential for maintaining a slit diaphragm structure and nephrocyte function [34]. Therefore, we examined whether the genetically modified Pink1–Park pathway affected the Rab GTPases we had identified as most important for the nephrocyte slit diaphragm [34]. We found a significant reduction in all three—Rab5 (early endosome), Rab7 (late endosome), and Rab11 (recycling endosome)—after silencing or overexpressing *Pink1*, *park*, or *Marf* (Figure 5). These results highlight the critical role of mitochondrial dynamics in endocytic membrane trafficking. Disrupted trafficking following Pink1–Park pathway genetic modification, likely underlies the associated altered slit diaphragm filtration structure and impaired nephrocyte function. 

### 3.4. Pink1–Park-Mediated Defective Mitochondrial Dynamics Lead to Induced Cellular Protein Aggregation and Increased ROS 

**Figure 6:**
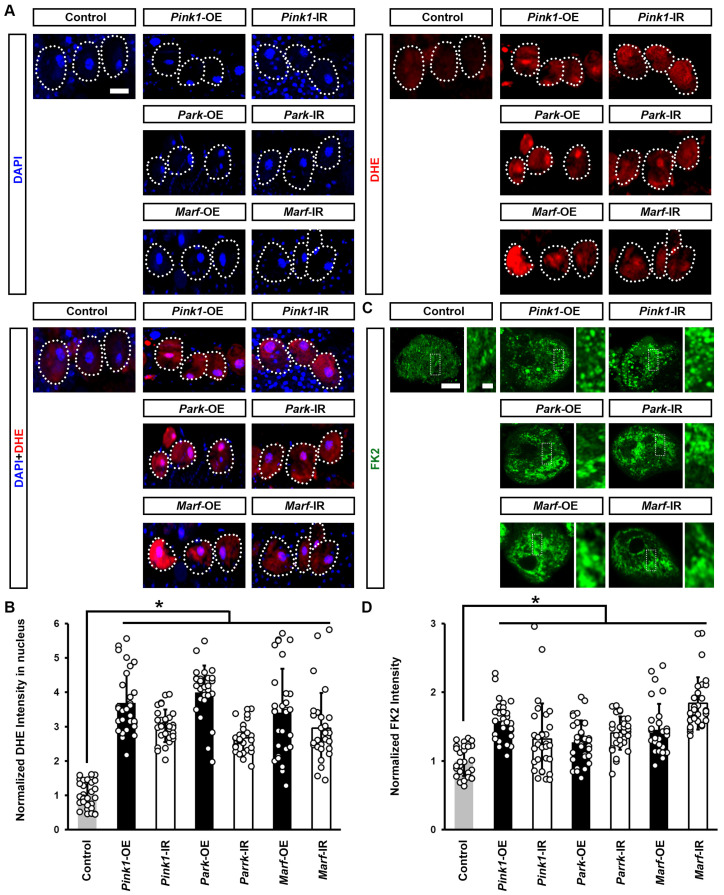
Defective mitochondrial dynamics in nephrocytes induced protein aggregation and increased ROS: (**A**–**C**) Reactive oxygen species (ROS) and protein aggregation in *Drosophila* nephrocytes (female, 4-day-old) overexpressing (OE) or inhibiting (RNAi; IR) *Pink1*, *park*, or *Marf*. Control, *Dot* > *w*^1118^. (**A**) Dihydroethidium (DHE; red) labels reactive oxygen species (ROS); and, DAPI (blue) was used to visualize the nuclei in nephrocytes. Scale bar = 25 µm. (**B**) Quantitation of DHE fluorescence in *Pink1*/*park*/*Marf*-OE/IR nephrocytes. (**C**) Anti-ubiquitinylated proteins antibody, clone FK2 (green) was used to label protein aggregates in nephrocytes. Scale bar = 5 µm. Boxed areas are shown magnified to the right (scale bar: 1 μm). (**D**) Quantitation of FK2 fluorescence in *Pink1*/*park*/*Marf*-OE/IR nephrocytes, relative to control. N = 30 nephrocytes from 6 flies, per group per assay. Results are presented as mean ± SD. Statistical significance (*) is defined as *p* < 0.05.

To understand why defective mitochondrial dynamics caused *Drosophila* nephrocyte damage, we further investigated cellular changes induced by impaired endocytic membrane trafficking. Reactive oxygen species (ROS) production and oxidative stress have been implicated in mitochondrial dysfunction [35]. We examined the effect of silencing or overexpressing *Pink1*, *park*, or *Marf* on ROS levels in *Drosophila* nephrocytes. Dihydroethidium (DHE) was used as a redox indicator [23,36]. Reduced DHE fluoresces blue, but it shifts to red fluorescence upon oxidation, and intercalates into DNA. We observed a significant increase in DHE red fluorescence intensity in the nephrocyte nucleus after silencing or overexpressing *Pink1*, *park*, or *Marf* (Figure 6A,B). Furthermore, these nephrocytes contained aggregates of ubiquitinylated proteins, another indicator of defective endocytic membrane trafficking and autophagy (Figure 6C,D).

### 3.5. Defective Endocytosis Leads to Reduced Nephrocyte Function, Induced Cellular Protein Aggregation, and Increased ROS 

**Figure 7:**
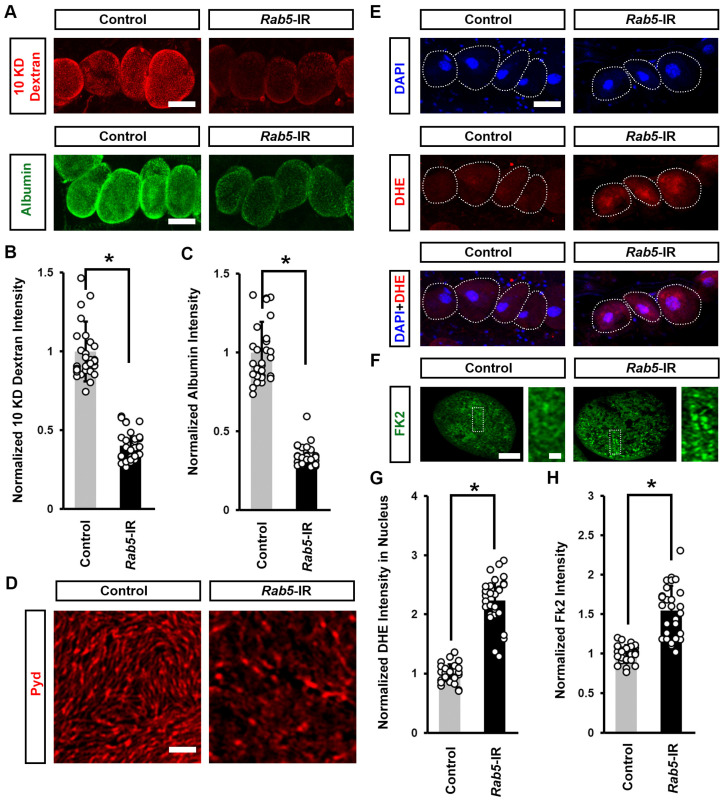
Defective endocytosis reduced nephrocyte function, induced protein aggregation, and increased ROS: (**A**–**H**) Function, protein aggregation, and ROS in *Drosophila* nephrocytes (female, 4-day-old) inhibiting (RNAi; IR) *Rab5*. Control, *Dot* > *w*^1118^. (**A**) 10 kDa dextran (red) and albumin (green) uptake by nephrocytes. Scale bar = 25 µm. (**B**) Quantitation of 10 kDa dextran fluorescence in *Rab4*-IR nephrocytes. (**C**) Quantitation of albumin fluorescence in *Rab5*-IR nephrocyte. (**D**) Localization of slit diaphragm protein Polychaetoid (Pyd; red) by immunofluorescence in *Drosophila* nephrocytes. Imaged at nephrocyte surface. Scale bar = 1 µm. (**E**) Dihydroethidium (DHE; red) labels reactive oxygen species (ROS); and, DAPI (blue) was used to visualize the nuclei in nephrocytes. Scale bar = 25 µm. (**F**) Quantitation of DHE fluorescence in *Rab5*-IR nephrocytes. (**G**) Anti-ubiquitinylated proteins antibody, clone FK2 (green) was used to label protein aggregates in nephrocytes. Scale bar = 5 µm. Boxed areas are shown magnified to the right (scale bar: 1 μm). (**H**) Quantitation of FK2 fluorescence in *Rab5*-IR nephrocytes, relative to control. N = 30 nephrocytes from 6 flies, per group per assay. Results are presented as mean ± SD. Statistical significance (*) is defined as *p* < 0.05.

To understand whether induced cellular protein aggregation and increased ROS were caused by defective endocytosis induced by mitochondrial dysfunction, we knocked down the endocytic-associated gene *Rab5* in flies. We noted a significant decline in both 10 kDa dextran and albumin intensity when silencing *Rab5* in the nephrocytes (Figure 7A–C), indicating nephrocyte functional decline. We further observed a disrupted slit diaphragm localization pattern at the nephrocyte surface (Figure 7D) and a significant increase in DHE red fluorescence intensity in the nephrocyte nucleus and protein aggregation after silencing *Rab5* (Figure 7E–H). Previous studies suggest that defective endocytosis can impair autophagy and lead to protein aggregation [37,38]. Protein aggregation in cells has been linked to increased ROS levels [39,40]. Together, these findings suggest that defects in endocytosis caused by mitochondrial dysfunction can lead to protein accumulation, which in turn may increase ROS levels.

**Figure 8:**
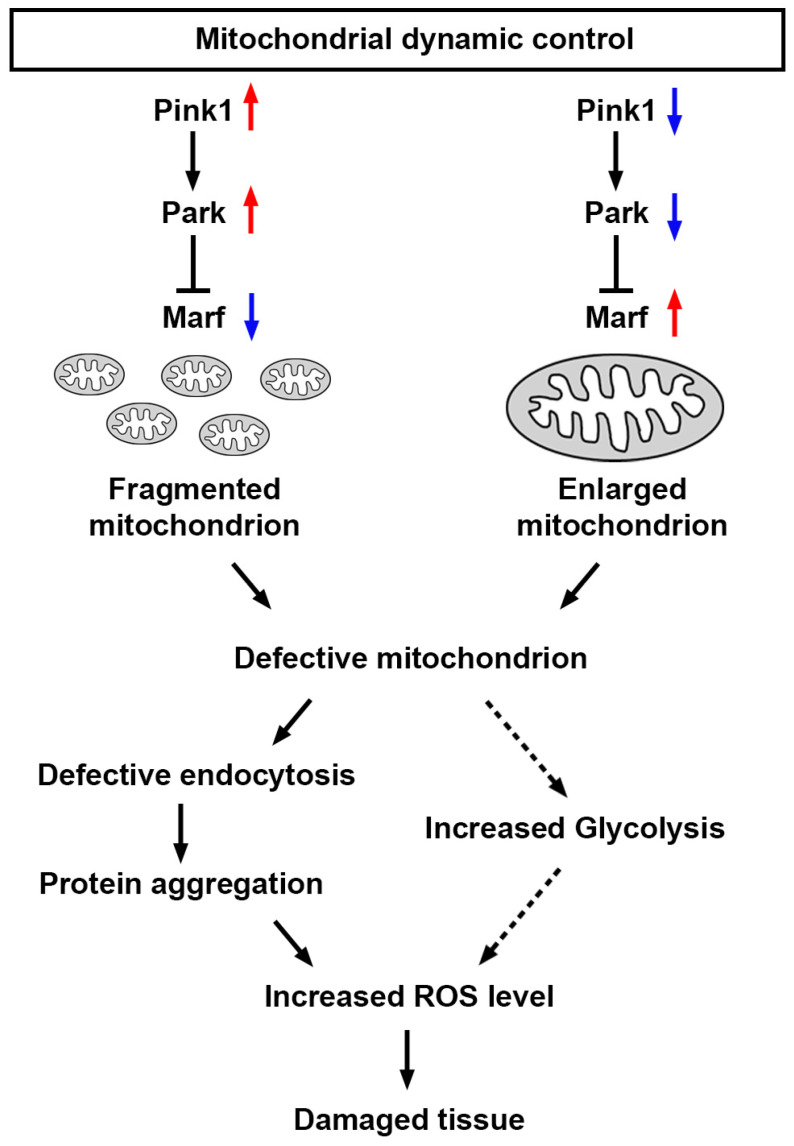
Model of nephrocyte damage mediated by mitochondrial dynamics Schematic representation of the regulation of mitochondrial dynamics. Mitochondrial dynamics can be disrupted by the genetic modification of the Pink1–Park pathway (*Pink1*/*park*/*Marf* overexpression or silencing; red upward arrow indicates increase; blue downward arrow indicates decrease). The resulting changes in mitochondrial morphology lead to mitochondrial dysfunction, which impairs the endocytic trafficking pathway, resulting in protein aggregation, and increased reactive oxygen species (ROS), culminating in further tissue damage. Marf, Mitochondrial assembly regulatory factor; Park, Parkin; Pink1, PTEN-induced putative kinase 1.

Altogether, these findings demonstrate that a defective Pink1–Park pathway—through silencing or overexpressing *Pink1*, *park*, or *Marf*—leads to altered mitochondrial dynamics, which disrupts endocytosis mechanisms, which induces protein aggregation and further increases cellular ROS levels, which likely plays a role in the abnormal uptake function observed in nephrocytes (Figure 8). However, mitochondrial dysfunction can also enhance glycolysis [41,42], leading to increased production of pyruvate, which can be converted to lactate. This process regenerates NAD+ from NADH and contributes to increased ROS levels.

## 4. Discussion

The *Drosophila* nephrocyte, a podocyte-like cell, features a filtration slit diaphragm and exhibits remarkable structural and functional similarities with the mammalian podocyte [19,43,44]. Both nephrocytes and podocytes form highly specialized filtration barriers [39]. Filtered molecules that pass through these filters in nephrocytes enter the lacuna channel, analogous to the urinary space in the Bowman’s capsule of human kidneys. While in human kidneys, filtered molecules undergo reabsorption or excretion, in nephrocytes, these molecules are absorbed and processed by the cells. Proteins are degraded for amino acid recycling, and waste is detoxified and stored in vacuoles. Although there are differences in processing post-filtration, nephrocytes perform a true filtration function that closely resembles that of podocytes [45]. Since being identified more than a decade ago, the *Drosophila* nephrocyte has emerged as an invaluable model for exploring development processes and kidney diseases [32,44,46].

Here, we explored the roles of mitochondria in *Drosophila* nephrocytes, focusing on how molecular regulators of mitochondrial dynamics through fission and fusion pathways maintain mitochondrial function. These pathways are highly conserved from *Drosophila* to humans [4,47,48,49]. We found that genetic manipulation of the Pink1–Park pathway disrupted mitochondrial dynamics, leading to abnormalities in the slit diaphragm and functional defects in the nephrocytes. Additionally, mitochondrial dysfunction in nephrocytes triggered defects in endocytosis, protein aggregation, increased levels of ROS, and, ultimately, cell damage (Figure 7). Our findings illustrate that dysfunction in mitochondrial dynamics in *Drosophila* nephrocytes leads to various functional defects, underscoring the critical role of mitochondria in nephrocyte development and kidney diseases. This research provides a valuable model for studying the mechanisms underlying mitochondrial-related kidney diseases and offers insights into potential therapeutic strategies.

Numerous genetic mutations have been linked to monogenic forms of nephrotic syndrome (NS), including several genes involved in the mitochondrial CoQ pathway [20,23,50]. However, significant gaps in understanding the roles of these genes in kidney cell biology and disease mechanisms remain. For instance, a deficiency in CoQ2, a component of the CoQ pathway, results in mitochondrial dysfunction and mislocalization of slit diaphragms [45,46]. Our current study suggests this might be due to endocytosis defects. Additionally, mitochondrial dysfunction has been implicated in diabetic nephropathy [51,52]. In previous studies using *Drosophila*, chronic exposure to high dietary sucrose mimicked several features of diabetic nephropathy [24,25,53]. Chronic sucrose intake also led to morphological abnormalities in the mitochondria of *Drosophila* nephrocytes, linked to defects in mitochondrial fission [24]. Genetic or pharmacological intervention at the Pink1–Park pathway attenuated the mitochondrial dysfunction and slowed the decline in nephrocyte function in the high-sucrose-fed flies [24]. Here, we showed the trajectory from disrupted mitochondrial dynamics to damaged tissue, through reduced endocytic trafficking, associated increased protein aggregates, and increased ROS (Figure 7). These, and previous, findings highlight the critical role of mitochondrial health in kidney function and provide a valuable framework for exploring therapeutic interventions in mitochondrial-related kidney diseases.

## Data Availability

The original contributions presented in the study are included in the article, and further inquiries can be directed to the corresponding authors.
